# Cardiometabolic multimorbidity in Mexican adults: a cross-sectional analysis of a national survey

**DOI:** 10.3389/fmed.2024.1380715

**Published:** 2024-09-03

**Authors:** Marcela Agudelo-Botero, Claudio A. Dávila-Cervantes, Liliana Giraldo-Rodríguez

**Affiliations:** ^1^Centro de Investigación en Políticas, Población y Salud, Facultad de Medicina, Universidad Nacional Autónoma de México, Mexico City, Mexico; ^2^Facultad Lationoamericana de Ciencias Sociales, Sede México, Mexico City, Mexico; ^3^Instituto Nacional de Geriatría, Mexico City, Mexico

**Keywords:** multimorbidity, cardiometabolic diseases, adulthood, prevalence, latent class, factor analysis, relative risk

## Abstract

**Background:**

Cardiometabolic multimorbidity is a rising phenomenon that has been barely explored in middle-income countries such as Mexico.

**Objective:**

This study aimed to estimate the prevalence, associated factors, and patterns of cardiometabolic multimorbidity (2 and 3+ diseases) in Mexican adults (≥20 years old) by age group.

**Methods:**

A cross-sectional and secondary analysis of Mexico’s National Health and Nutrition Survey 2018–2019 was conducted. Information on eight diseases and other sociodemographic and health/lifestyle characteristics was obtained through self-reporting. Descriptive analyses were performed, and multinomial logistic regression models were calculated to identify the variables associated with cardiometabolic multimorbidity. Factor analysis and latent classes were estimated to determine disease patterns.

**Results:**

The prevalence of cardiometabolic multimorbidity for the total population study was 27.6% (13.7% for people with 2 diseases and 13.9% for people with 3+ diseases). By age group, the prevalence of 2+ diseases was 12.5% in the age group of 20–39 years, 35.2% in the age group of 40–59 years, and 44.5% in the age group of 60 years and older. The variables of depressive symptomatology and having functional limitations (1+) were statistically associated with cardiometabolic multimorbidity in almost all age groups. Patterns of cardiometabolic multimorbidity varied among adults in different age groups. Understanding the behavior of cardiometabolic multimorbidity at various stages of adulthood is a resource that could be used to design and implement intervention strategies. Such strategies should correspond to the population’s sociodemographic, health, and lifestyle characteristics and the specific disease patterns of each age group.

## 1 Introduction

Cardiometabolic multimorbidity is defined as the co-occurrence of two or more cardiometabolic disorders, such as diabetes type 2 (T2D), coronary heart disease (CHD), and stroke (1–3). Globally, the weighted mean age of individuals with multimorbidity (considering different combinations of cardiometabolic diseases and other chronic diseases) was estimated to be 56.9 years, with a standard deviation [SD] of 10.4 years (4). The prevalence was estimated to be 37.2% (95% CI: 34.9–39.4%); however, this percentage increased to 50% in people aged ≥60 years (4). The number of adults experiencing the concurrence of not only 2 diseases but also 3 or more diseases is rising every day, with cardiometabolic diseases being a leading contributor (5–8). Cardiometabolic multimorbidity is a growing health phenomenon closely associated with the demographic and epidemiological transition, in particular, due to the accelerated aging of the population and the change in morbidity and mortality patterns (1–3, 9). This increase has also been attributed to rapid urbanization, sedentary lifestyles, and unhealthy dietary practices (10). A review and meta-analysis that included 59 studies, most of them from high-income countries, estimated that the annual cost (in international dollar [I$] for 2021) of multimorbidity per person ranged from I$800 to I$150,000. However, this depends on disease combination, country, and other factors (11).

Although older adults are at increased risk for various combinations of multimorbidity (12–16), evidence suggests that multimorbidity is a rising problem in younger adults (4, 5, 17, 18). Thus, the patterns and characteristics of multimorbidity differ between younger and older adults (2). This could be explained by the socioeconomic conditions of older adults and a more significant accumulation of exposure to risk factors at older age (19). As a result, older adults are more likely to simultaneously present multiple chronic non-communicable diseases (CNCDs), mainly of the cardiometabolic type (12–14, 19). Regarding young adults, it has been suggested that lifestyle changes have led to more cardiometabolic disorders (16, 20), as well as a higher prevalence of depression and anxiety (21, 22).

Several authors have consistently reported that different multimorbidity clusters (including cardiometabolic multimorbidity) are more prevalent in low- and middle-income countries (23–26). Therefore, it has become a challenge for healthcare systems that have traditionally focused on disease-specific management (27, 28). Additionally, it has been documented that multimorbidity has impacts at the individual, family, community, and institutional levels (4–6, 23). Multimorbidity is also linked to worse health outcomes, including disabilities, loss of quality of life, risk of recurrent hospitalizations, and premature death (1, 2, 6, 9, 14, 15, 19, 28). In Mexico, it was estimated that in 2022, approximately 3.9 years of life expectancy would be lost due to cardiometabolic diseases (between 0 and 85 years), including heart disease, stroke, T2D, or hypertension-related diseases (29). Due to the high mortality burden of cardiometabolic diseases, the Mexican government declared T2D, overweight, and obesity a national epidemic in 2016 (30).

To respond to this complex scenario, some researchers agree that clear epidemiological information that reflects the particularities of population subgroups is required (18, 21). This is because there is a scarcity of information on the magnitude and patterns of cardiometabolic multimorbidity worldwide (4, 5). The objective of this study was to estimate the prevalence, associated factors, and patterns of cardiometabolic multimorbidity (2 and 3+ diseases) in Mexican adults (≥20 years old) by age group. The fact that the above causes are the most prevalent and represent the highest costs for the national health system was considered (31).

## 2 Materials and methods

A cross-sectional and secondary analysis of Mexico’s National Health and Nutrition Survey 2018–2019 (ENSANUT, for its acronym in Spanish) (32) was conducted. Its objective was to update the frequency, distribution, and trend of health and nutrition conditions and their social determinants and to study coverage, targeting, perceived quality, and user satisfaction with health and nutrition programs and services. This probabilistic survey provides information at the national and sub-national levels (32 states) (32). This study sample comprised 16,835 adults, representing 78,463,734 Mexicans aged ≥20.

### 2.1 Dependent variable

Cardiometabolic multimorbidity groups were without multimorbidity (0 or 1 disease) or with multimorbidity (2 or more diseases). Cardiometabolic multimorbidity was classified into two groups: 2 diseases and 3+ diseases. A total of eight cardiometabolic diseases were considered for this study: obesity, T2D, high blood pressure, hypercholesterolemia, hypertriglyceridemia, acute myocardial infarction, angina, and heart failure. Identification of these diseases was compiled through self-reporting through the questions: “Has a physician ever told you that you have diabetes (or high blood sugar), high blood pressure, high cholesterol, high triglycerides?”; “Have you ever had severe chest pain, sweating, shortness of breath, or severe discomfort that lasted half an hour?” “Has a physician ever told you that you have or had a myocardial infarction or heart attack; angina pectoris (chest pain or discomfort, which regularly goes away spontaneously with rest or medication), heart failure (weakening of the heart’s pumping ability, causing edema of the feet, ankles, and legs, tiredness and shortness of breath)?”; “Has a physician/dietitian/nutritionist ever told you that you have or had obesity?” Responses to these questions were dichotomized (no/yes) (32). Data were stratified by age group (20–39/40–59/60+).

### 2.2 Covariates

Regarding the factors associated with cardiometabolic multimorbidity and considering the availability of these data in the ENSANUT, the following covariates were included based on the literature (2, 10, 19, 33).

#### 2.2.1 Sociodemographic

The following individual factors were included:

- Sex (men/ women).
- Educational level (elementary or lower education/secondary school/high school or higher education).
- Locality (rural [<2,500 inhabitants]/urban ≥2,500 inhabitants]).
- Socioeconomic level (low/medium–low, medium–high/high). This was determined using information on housing construction materials, the number of household appliances, and the number of electrical appliances. The principal components method, generated from a polychoric correlation matrix, created the index (32).
- Region (North [Baja California, Baja California Sur, Coahuila, Chihuahua, Durango, Nuevo León, San Luis Potosí, Sinaloa, Sonora, and Tamaulipas y Zacatecas; Center [Aguascalientes, Colima, Guanajuato, Hidalgo, Jalisco, Michoacán, Morelos, Nayarit, Querétaro, and Mexico City/Adjacent urban towns of the Estado de México; and South [Campeche, Hidalgo, Chiapas, Guerrero, Oaxaca, Puebla, Tlaxcala, Quintana Roo, Tabasco, Veracruz, and Yucatán]) (32).

#### 2.2.2 Health/lifestyles

The following factors were considered:

- Social security (none/secretariat of health/Mexican Social Security Institute [IMSS, for its acronym in Spanish]/Government Worker’s Social Security and Services Institute [ISSSTE, for its acronym in Spanish]/private insurance/other). The Mexican health system has three main components: employment-based social insurance schemes, public assistance services for the uninsured, and a private sector. Social insurance is centrally administered, while state and federal authorities manage coverage for the uninsured (34).
- Depressive symptomatology: The depressive symptoms in the past week were evaluated by using seven questions with four answer options: “rarely or never (less than one day),” “seldom or sometimes (from 1 to 2 days),” “a significant number of times (from 3 to 4 days),” and “all or most of the time (from 5 to 7 days).” Each question got a score between 0 and 3. The total score ranged from 0 to 21, where a higher score represented a more significant occurrence of depressive symptoms. Nine points or higher were set as the cut-off point to indicate the symptomatology of moderate or severe depression (35).
- Functional limitations. These limitations were determined based on the difficulties that people reported in walking, moving (or using arms and hands), learning, remembering, concentrating, communicating, or performing daily activities (36) (no/1/2+).
- Tobacco consumption: (never/currently no/currently yes [last 30 days]) (32).
- Alcohol consumption (never/currently no/currently yes). Current alcohol consumption was defined as the percentage of individuals who reported consuming at least one standard alcoholic beverage in the past 12 months. A standard drink contains approximately 13 g of pure alcohol (330 mL of beer, 140 mL of wine, or 70 mL of spirits). Alcohol consumption frequency was categorized by day, week, month, or year (37).

### 2.3 Analysis

A descriptive analysis of the population’s general characteristics and the correlation between cardiometabolic multimorbidity and covariates was identified. For this purpose, Pearson’s chi-square test of independence (χ^2^) was used. Then, multinomial regression models were estimated, and relative risk (RR) (38) was obtained to identify the factors associated with having 2 and 3+ diseases (the population without cardiometabolic multimorbidity was taken as a reference). The sociodemographic co-variables were considered as possible confounding factors. The significance level was two-sided, with *p* < 0.05 and a 95% CI.

Subsequently, latent class analysis (LCA) with its respective 95% CI was calculated to analyze how people are classified by type of cardiometabolic disease. LCA is a statistical procedure to identify qualitatively different subgroups within populations with specific outward characteristics (39). Subgroups are referred to as latent groups (or classes). LCA uses study participants’ responses to categorical indicator variables to detect latent groups. Each class represents a typology that can help researchers and practitioners understand commonalities and differences across individuals that have implications for practice and future research (39, 40). Three groups were established by evaluating the best-fitting and most parsimonious model based on the Bayesian information criterion (BIC) and Akaike’s information criterion (AIC) (40). The probability of individuals belonging to each of these groups was estimated based on the probabilities of having each cardiometabolic disease, where a low probability was considered “healthy,” a medium probability as “moderately healthy,” and a high probability as “unhealthy.”

Finally, a factor analysis (FA) was conducted to identify underlying co-occurrence patterns among various cardiometabolic diseases (41). FA is a technique used to identify observed data structures and reveal the underlying constructs that give rise to observed phenomena. The technique identifies and examines clusters of inter-correlated variables. This method uses sample data to model the population covariance matrix of a set of variables (42). Multimorbidity patterns were determined using exploratory FA based on polychoric correlations. We used all the available chronic health conditions coded as dichotomous variables. We applied the principal factors method and an oblique promax rotation. The number of factors was determined based on those with eigenvalues greater than 1 (Supplementary Table S1). A factor loading greater than 0.30 was considered significant and was used as the criterion for assigning each cardiometabolic disease to the different factors. The Kaiser–Meyer–Olkin (KMO) measure and Bartlett’s test (43) were employed as indicators of data adequacy and variable correlations, respectively, to assess the model’s goodness of fit.

Stata^®^ 17 was used for data processing (44), and the effect of the survey’s complex sampling design (*svy*) was considered.

## 3 Results

**Figure 1:**
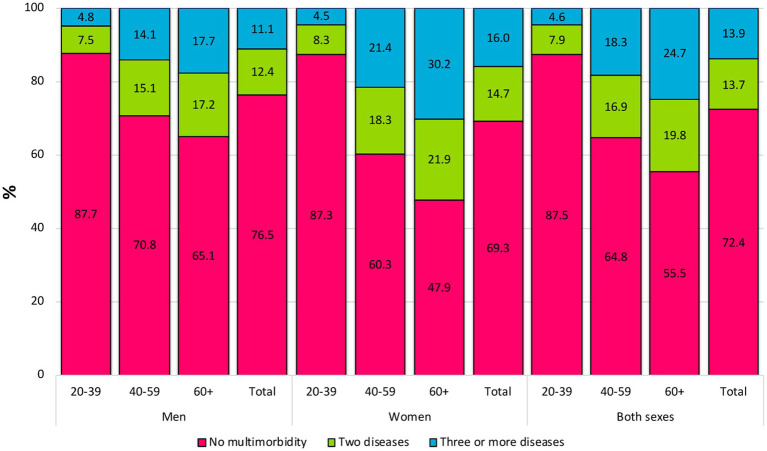
Prevalence of cardiometabolic multimorbidity by sex and age groups among Mexican adults (≥20 years).

The prevalence of cardiometabolic multimorbidity for the total population study was 27.6%: 13.7% for people with 2 diseases and 13.9% for people with 3+ diseases. In Figure 1, the prevalence of cardiometabolic multimorbidity is shown by age group (2+ diseases): 20–39 years (12.5%), 40–59 years (35.2%), and 60+ years (44.5%). Considering both sexes, the prevalence of cardiometabolic multimorbidity due to 3+ diseases was higher than that due to 2 diseases, both in the age groups of 40–59 years and 60+ years. The prevalence of cardiometabolic multimorbidity (2+ diseases) was higher among women (30.7%) than among men (23.5%). For women of all age groups, the prevalence of 3+ diseases was higher than that of 2 diseases (except for the age group of 20–39 years). On the other hand, the prevalence of 2 diseases was higher than that of 3+ diseases for men (except for the age group of 60+ years).

Table 1 shows the study population’s sociodemographic and health/ lifestyle characteristics based on their cardiometabolic multimorbidity status. Overall, the population was predominantly women of younger age (particularly for the group without cardiometabolic multimorbidity). Most of them lived with a partner of medium–low or low socioeconomic status and lived in urban areas. Approximately 60% of the total sample had no social security or used the services provided by the Secretariat of Health. Furthermore, 15.2% had depressive symptoms, which increased to 18.1 and 28.1% for people with 2 and 3+ diseases, respectively. Most of the people had no functional limitations (60%), but 53% of the people with 2 diseases and 65.6% of the people with 3+ diseases; 16% of adults were smokers, and 34% consumed alcohol. In the chi-square tests, statistically significant differences were found in the sociodemographic and health/lifestyle characteristics among almost all covariates, except for marital status.

**Table 1:** Sociodemographic and health characteristics of the study population by cardiometabolic multimorbidity status (*n* = 16,835).

Covariates/Category		Multimorbidity (n)				χ^2^
		No multimorbidity	Two diseases	Three or more diseases	Total	
		12,073	2,343	2,419	16,835	
		percentual distribution (%)				
Sociodemographic						
Sex	Men	45.0	38.4	33.8	42.5	0.000
	Women	55.0	61.6	66.2	57.5	
Age group	20–39 years	50.8	24.3	13.9	42.0	0.000
	40–59 years	32.9	45.0	48.3	36.7	
	60+ years	16.3	30.7	37.8	21.3	
Educational level	Elementary school or lower education	32.6	44.2	48.8	36.4	0.000
	Secondary school	31.0	28.2	29.3	30.4	
	High school or higher education	36.4	27.6	21.9	33.2	
Socioeconomic status	Low	24.6	20.0	15.9	22.8	0.000
	Medium–low	51.4	51.8	54.7	51.9	
	Medium–high	17.5	21.0	22.0	18.6	
	High	6.5	7.2	7.4	6.7	
Locality	Rural	30.0	24.1	21.4	28.0	0.000
	Urban	70.0	75.9	78.6	72.0	
Region	North	17.5	19.7	21.8	18.4	0.000
	Center	37.5	31.2	28.8	35.4	
	Mexico City	12.0	15.4	13.2	12.6	
	South	33.0	33.7	36.2	33.6	
Health/lifestyles						
Social security	None	17.3	13.3	9.5	15.6	0.000
	Secretariat of Health	43.1	36.8	35.2	41.1	
	IMSS	31.7	36.9	43.2	34.0	
	ISSSTE	5.6	9.2	8.7	6.6	
	Private	1.4	2.1	2.0	1.6	
	Others	0.9	1.7	1.4	1.1	
Depressive symptoms	No	87.8	81.9	71.9	84.8	0.000
	Yes	12.2	18.1	28.1	15.2	
Functional limitations	No	67.4	47.0	34.4	60.0	0.000
	One	18.4	26.3	25.7	20.5	
	Two or more	14.2	26.7	39.9	19.5	
Tobacco consumption (n = 16,799)	Never	62.7	64.6	63.8	63.1	0.000
	Current no	19.6	21.8	25.0	20.6	
	Current yes	17.7	13.6	11.2	16.3	
Alcohol consumption	Never	34.9	38.4	40.0	36.1	0.000
	Current no	29.0	32.3	32.3	29.9	
	Current yes	36.1	29.3	27.7	34.0	

IMSS, Instituto Mexicano del Seguro Social; ISSSTE, Instituto de Seguridad y Servicios Sociales de los Trabajadores del Estado; χ^2^: chi-square test; *Missing data were “no response”.

Table 2 includes the multinomial logistic regression models for 2 and 3+ cardiometabolic diseases. The sociodemographic and health/ lifestyle covariates had differential behavior among the age groups. However, in general, both depressive symptoms and functional limitations were the associated variables for all age groups (except for people aged 20–39 years having a functional limitation and with 3+ diseases). Having 2+ functional limitations in the youngest age group was associated with an increased RR of 3.17 for those with 2 cardiometabolic diseases. Current not consumption alcohol was associated with a decreased relative risk (RR) of 0.79 for individuals with three or more diseases. The association of RR had a statistically significant decrease among adults aged 40–59 years with secondary school, high school, or higher education levels who had 2 diseases. Additionally, a significant decrease was observed for those with high school or higher education levels who had 3+ diseases. For the same age group, having social security was also associated with an increase in the RR of having 3+ diseases (which was lower for those with private insurance) compared to those who reported not having any social security. In turn, more statistically significant variables were associated with an increased RR of having cardiometabolic multimorbidity—especially 3+ diseases—in the older adult group (60 years and older). Social security was associated with a higher RR of having 2 diseases. In addition, the fact of being a woman increased this RR of having 3+ diseases to 2.98. Such RR also increased for people of all socioeconomic strata.

**Table 2:** Factors associated with cardiometabolic multimorbidity in Mexican adults by age groups and numbers of diseases (2 and 3+) (*N* = 4,762).

Covariates/Category	Relative risk (RR) (95% IC)						
	20–39 years		40–59 years		60 years or more		
	Two diseases	Three or more diseases	Two diseases	Three or more diseases	Two diseases		Three or more diseases
Sociodemographic							
Sex							
Men†	1	1	1	1	1		1
Women	1.16 (0.94–1.43)	0.91 (0.70–1.18)	1.31 (1.10–1.55)**	1.47 (1.24–1.75)*	1.71 (1.37–2.13)*		2.98 (2.40–3.70)*
Educational level							
Elementary school or lower education†	1	1	1	1	1		1
Secondary school	1.42 (1.06–1.91)**	0.92 (0.65–1.31)	0.79 (0.67–0.94)**	0.93 (0.79–1.10)	0.90 (0.69–1.17)		1.26 (0.99–1.59)
High school or higher education	1.20 (0.89–1.62)	0.72 (0.50–1.04)	0.78 (0.63–0.95)**	0.75 (0.61–0.93)**	0.87 (0.64–1.20)		1.07 (0.79–1.44)
Socioeconomic status							
Low†	1	1	1	1	1		1
Medium–low	1.11 (0.84–1.48)	1.45 (0.98–2.14)	0.96 (0.76–1.22)	1.25 (0.98–1.59)	1.35 (1.01–1.80)**		2.35 (1.77–3.13)*
Medium–high	1.23 (0.84–1.79)	1.48 (0.89–2.45)	1.02 (0.75–1.39)	1.23 (0.90–1.68)	1.37 (0.95–1.98)		2.73 (1.91–3.91)*
High	0.74 (0.43–1.29)	1.63 (0.88–3.04)	1.15 (0.80–1.65)	1.43 (0.99–2.06)	0.96 (0.60–1.54)		1.65 (1.04–2.61)**
Locality							
Rural†	1	1	1	1	1		1
Urban	1.17 (0.90–1.52)	1.46 (1.02–2.08)**	1.23 (0.99–1.53)	1.69 (1.36–2.10)*	1.22 (0.95–1.58)		0.87 (0.68–1.11)
Region							
North†	1	1	1	1	1		1
Center	0.79 (0.60–1.03)	0.83 (0.59–1.18)	0.76 (0.62–0.94)**	0.73 (0.59–0.89)	0.86 (0.67–1.09)		0.64 (0.51–0.81)*
Mexico City	0.85 (0.59–1.21)	0.81 (0.50–1.32)	1.25 (0.99–1.58)	0.85 (0.67–1.09)	0.76 (0.57–1.02)		0.56 (0.42–0.74)*
South	1.08 (0.82–1.43)	1.58 (1.11–2.25)**	1.12 (0.90–1.39)**	1.27 (1.03–1.57)**	0.92 (0.70–1.20)		1.01 (0.79–1.29)
Health/lifestyles							
Social security							
None†	1	1	1	1	1		1
Secretariat of Health	0.93 (0.73–1.19)	0.96 (0.68–1.35)	0.88 (0.71–1.10)	1.59 (1.23–2.04)*	1.77 (1.27–2.47)*		1.44 (1.06–1.97)**
IMSS	0.77 (0.59–1.00)**	1.32 (0.94–1.85)	1.34 (1.07–1.67)**	2.39 (1.86–3.07)*	2.58 (1.87–3.56)*		2.33 (1.73–3.13)*
ISSSTE	1.18 (0.74–1.87)	1.34 (0.71–2.53)	1.76 (1.30–2.39)*	2.44 (1.74–3.43)*	2.45 (1.65–3.66)*		1.82 (1.26–2.65)*
Private	1.63 (0.83–3.22)	2.43 (1.06–5.55)	1.64 (1.01–2.66)**	1.64 (0.92–2.92)	2.16 (0.98–4.75)		3.08 (1.60–5.93)*
Others	2.47 (1.25–4.87)**	1.46 (0.42–5.09)	1.30 (0.65–2.60)	3.67 (1.96–6.88)*	6.62 (3.01–14.5)*		3.98 (1.75–9.06)*
Depressive symptoms							
No†	1	1	1	1	1		1
Yes	1.57 (1.22–2.03)*	2.42 (1.80–3.25)*	1.20 (0.99–1.46)	1.95 (1.65–2.32)*	1.13 (0.91–1.41)		1.74 (1.43–2.12)*
Functional limitations							
No†	1	1	1	1	1		1
One	1.59 (1.25–2.02)*	1.15 (0.82–1.61)	1.34 (1.14–1.58)*	1.94 (1.65–2.29)*	1.62 (1.27–2.06)*		1.74 (1.36–2.23)*
Two or more	3.17 (2.29–4.39)*	2.87 (1.93–4.28)*	1.29 (1.06–1.56)**	2.85 (2.39–3.40)*	1.69 (1.36–2.10)*		2.53 (2.03–3.15)*
Tobacco consumption							
Never†	1	1	1	1	1		1
Actually no	1.24 (0.97–1.58)	1.55 (1.13–2.13)**	1.14 (0.94–1.39)	1.29 (1.07–1.56)**	0.95 (0.76–1.20)		1.42 (1.14–1.76)*
Actually yes	1.17 (0.91–1.51)	1.26 (0.91–1.75)	0.81 (0.64–1.02)	0.78 (0.62–0.99)**	0.65 (0.47–0.90)**		0.69 (0.50–0.94)**
Alcohol consumption							
Never†	1	1	1	1	1		1
Actually no	1.24 (0.97–1.58)	0.79 (0.57–1.10)**	0.97 (0.81–1.16)	0.95 (0.79–1.14)	1.16 (0.93–1.44)		1.38 (1.11–1.70)*
Actually yes	1.17 (0.91–1.51)	0.83 (0.60–1.14)	0.96 (0.79–1.16)	1.00 (0.83–1.22)	1.02 (0.78–1.34)		1.45 (1.13–1.86)*
Constant	0.04 (0.03–0.07)	0.02 (0.01–0.04)	0.18 (0.13–0.25)	0.04 (0.03–0.07)	0.07 (0.04–0.11)*		0.03 (0.02–0.05)

† Reference category; *p < 0.0001; **p < 0.05. IMSS, Instituto Mexicano del Seguro Social; ISSSTE, Instituto de Seguridad y Servicios Sociales de los Trabajadores del Estado.

Estimates of the probability of belonging to the groups are shown in Table 3: Class 1 (healthy), Class 2 (moderately healthy), and Class 3 (unhealthy). For the first age group (20–39 years old), Class 2 comprised people with a 77% probability of having obesity and a 35% probability of having high blood pressure. Class 3 comprised people with a 91% probability of having hypercholesterolemia, an 88% probability of having hypertriglyceridemia, and a 50% probability of having obesity. In the 40–59 age group, obesity occurred in the three latent classes, with probabilities ranging from 37 to 66%. Furthermore, high blood pressure played a notable role in Class 2 (89% probability), and hypercholesterolemia (90%) and hypertriglyceridemia (84%) stood out in Class 3. The older adult group had a 53, 33, and 81% probability of obesity, T2D, and high blood pressure, respectively. Likewise, the probability of having hypercholesterolemia (88%) and hypertriglyceridemia (77%), in addition to these diseases, was above 40% in Class 3. On the other hand, the latent classes demonstrated that, while most adults were classified in Class 1 as “healthy,” such a proportion increased as age increased (Table 3).

**Table 3:** Cardiometabolic disease probabilities per class in Mexican adults by age groups.

Cardiometabolic disease	Probability (95% CI)		
20–39 years			
	**Class 1** (86.5%)*	**Class 2** (7.4%)*	**Class 3** (6.1%)*
Obesity	0.28 (0.26–0.30)	**0.77 (0.60**–**0.87)**	**0.54 (0.47**–**0.60)**
Diabetes type 2	0.01 (0.00–0.01)	0.17 (0.04–0.11)	0.09 (0.06–0.14)
Hypertension	0.04 (0.03–0.05)	**0.35 (0.24**–**0.49)**	0.21 (0.16–0.26)
Hypercholesterolemia	0.02 (0.01–0.03)	0.23 (0.13–0.38)	**0.91 (0.18**–**1.00)**
Hypertriglyceridemia	0.02 (0.01–0.03)	0.23 (0.13–0.38)	**0.88 (0.35**–**0.99)**
Acute myocardial infarction	0.00 (0.00–0.00)	0.05 (0.02–0.10)	0.00 (0.00–0.04)
Angina	0.01 (0.00–0.01)	0.04 (0.02–0.09)	0.02 (0.01–0.05)
Heart failure	0.00 (0.00–0.01)	0.05 (0.02–0.10)	0.02 (0.01–0.04)

40–59 years			
	Class 1 (68.4%)*	Class 2 (11.1%)*	Class 3 (20.4%)*
Obesity	**0.37 (0.36**–**0.39)**	**0.66 (0.59**–**0.73)**	**0.52 (0.50**–**0.56)**
Diabetes type 2	0.06 (0.05–0.07)	0.29 (0.24–0.36)	0.28 (0.25–0.31)
Hypertension	0.06 (0.04–0.10)	**0.89 (0.72**–**0.97)**	**0.38 (0.36**–**0.42)**
Hypercholesterolemia	0.04 (0.04–0.06)	0.12 (0.07–0.21)	**0.90 (0.83**–**0.95)**
Hypertriglyceridemia	0.03 (0.02–0.04)	0.11 (0.07–0.19)	**0.84 (0.77**–**0.90)**
Acute myocardial infarction	0.00 (0.00–0.01)	0.04 (0.03–0.07)	0.03 (0.02–0-04)
Angina	0.00 (0.00–0.01)	0.04 (0.03–0.06)	0.02 (0.01–0.03)
Heart failure	0.00 (0.00–0.01)	0.04 (0.03–0.07)	0.02 (0.02–0.03)

60 years or more			
	**Class 1** (57.1%)*	Class 2 (19.4%)*	Class 3 (23.5%)*
Obesity	0.21 (0.19–0.24)	**0.53 (0.46**–**0.61)**	**0.42 (0.39**–**0.46)**
Diabetes type 2	0.16 (0.14–0.16)	**0.33 (0.28**–**0.39)**	**0.41 (0.38**–**0.45)**
Hypertension	0.20 (0.16–0.27)	**0.81 (0.71**–**0.89)**	**0.66 (0.63**–**0.70)**
Hypercholesterolemia	0.06 (0.05–0.09)	0.06 (0.01–0.26)	**0.88 (0.79**–**0.94)**
Hypertriglyceridemia	0.02 (0.02–0.04)	0.03 (0.01–0.19)	**0.77 (0.66**–**0.85)**
Acute myocardial infarction	0.00 (0.00–0.02)	0.09 (0.07–0.14)	0.04 (0.04–0.07)
Angina	0.00 (0.00–0.01)	0.06 (0.05–0.10)	0.04 (0.03–0.06)
Heart failure	0.00 (0.00–0.02)	0.10 (0.07–0.15)	0.05 (0.04–0.07)

*Distribution of persons in each class. Conditional probabilities ≥ 30% in bold to facilitate interpretation.

In the FA, diseases were clustered into two factors across different age groups (Table 4). In the 20–39 age group, Factor 1, which includes obesity, T2D, hypertension, hypercholesterolemia, and hypertriglyceridemia, reflected a clear grouping of metabolic conditions, while Factor 2 encompassed cardiopathies such as acute myocardial infarction, angina, and heart failure. In the 40–59 age group, Factor 1 was dominated by cardiopathies and Factor 2 by metabolic diseases. In the 60+ age group, although metabolic diseases remained grouped in Factor 1, hypertension was associated with Factor 2 and cardiopathies, indicating a more significant interrelation of these conditions in older people. The KMO coefficient was marginally adequate for the data from the age groups of 20–39 years and 60+ years, while it was moderately adequate for the 40–59 age group. Bartlett’s test obtained a *p*-value of ≤0.000 in all age groups.

**Table 4:** Factor analysis of cardiometabolic multimorbidity in Mexican adults by age groups.

Age groups	Cardiometabolic disease	Factor 1	Factor 2
20–39	Obesity	**0.433**	−0.033
	Diabetes type 2	**0.472**	0.026
	Hypertension	**0.419**	0.166
	Hypercholesterolemia	**0.848**	0.009
	Hypertriglyceridemia	**0.847**	0.008
	Acute myocardial infarction	0.089	**0.696**
	Angina	0.008	**0.703**
	Heart failure	−0.024	**0.808**
40–59	Obesity	0.073	0.183
	Diabetes type 2	0.053	**0.408**
	Hypertension	**0.414**	0.316
	Hypercholesterolemia	−0.029	**0.901**
	Hypertriglyceridemia	0.009	**0.895**
	Acute myocardial infarction	**0.790**	0.021
	Angina	**0.715**	−0.023
	Heart failure	**0.836**	−0.018
60+	Obesity	0.195	0.132
	Diabetes type 2	**0.349**	−0.018
	Hypertension	0.335	**0.392**
	Hypercholesterolemia	**0.898**	−0.041
	Hypertriglyceridemia	**0.891**	0.004
	Acute myocardial infarction	−0.064	**0.717**
	Angina	−0.007	**0.770**
	Heart failure	0.009	**0.746**

Values in bold show clusters of inter-correlated cardiometabolic diseases. Rotation: Oblique promax (Kaiser off).

## 4 Discussion

In this study, we found that cardiometabolic multimorbidity affects approximately one-third of the sample population, increasing progressively with age and being more prevalent among women than among men. Depressive symptoms and functional limitations were the two variables most strongly associated with cardiometabolic multimorbidity across all age groups. Distinct patterns of cardiometabolic multimorbidity emerged across different groups, with obesity consistently appearing as a common factor in the latent class analysis. The diseases predominantly clustered into two groups that varied by age.

As observed in other countries, cardiometabolic multimorbidity is becoming more prevalent among young adults, but its prevalence is higher among older adults (5, 8, 9). The data obtained in this study are not precisely comparable to those of other studies mainly because the number and nature of the diseases analyzed are different (1, 3, 8, 15, 33, 45). Despite this, cardiometabolic multimorbidity in Mexican people aged ≥20 years (27.6%) was higher than that reported in the United States (14.4%) (3). It was also higher than that reported (13.7%) in a multi-cohort study that clustered information from 14 countries, where adults aged ≥50 years were included (16). Nevertheless, it should be noted that obesity was not considered part of cardiometabolic diseases in the two studies (3, 16). This fact could explain the value obtained for Mexico. Other studies on multimorbidity, including a broader range of diseases such as cancer and arthritis, suggest that cardiometabolic diseases have a notable impact on multimorbidity (3, 8, 16, 19). Therefore, multimorbidity prevalence is lower in countries with less frequent cardiometabolic diseases.

The health profile of Mexican people is increasingly complex because cardiometabolic diseases begin to occur at a young age (20). Early-onset diseases are associated with poor outcomes in old age (46). According to the World Health Organization (WHO), adult health is determined by behaviors initiated during adolescence and young adulthood, when lifestyles take root (47). The expansion of morbidity leads to people living with coexisting chronic diseases from an early age, which implies a more significant healthcare burden (48). There is increasingly more information and understanding of multimorbidity in older adults (12, 13, 19). Nevertheless, more knowledge and a deeper understanding of the dynamics, risk factors, and sequelae in young and middle-aged adults are required (17, 18, 21, 22, 33).

Mexicans with cardiometabolic multimorbidity are mainly people with an elementary school or lower educational level, a medium–low socioeconomic level, and residents of the southern region (characterized by concentrating the states with the highest levels of poverty and marginalization in the country). Furthermore, many people do not have social security or receive limited services from the Ministry of Health. Similarly, we found that the factors associated with cardiometabolic multimorbidity in Mexican adults correspond to those in other studies (19, 46, 49, 50). Mainly, we observed that the main covariates related to cardiometabolic multimorbidity in almost all age groups with 2 and 3+ diseases were depressive symptoms and functional limitations.

A meta-analysis conducted in 43 low- and middle-income countries observed a significant positive correlation between depression and multimorbidity, with an odds ratio (OR) of 3.26 (95% CI, 2.98–3.57) (51). It is known that there is a bidirectional relationship between depression and CNCDs, although the mechanisms that connect them are unclear so far (52). In this regard, some authors have reported the link between depression and CNCDs, such as obesity and T2D, in Mexican adults (53). People with multimorbidity and depression experience more disabilities, pain, and loss of control over important aspects of their lives than those without either of these diseases (52).

Moreover, cardiometabolic multimorbidity can lead to more significant disabilities or functional limitations. This was observed in a study conducted in Ghana and South Africa, where the different cardiometabolic multimorbidity clusters were predictors of functional disability in adults aged 50 years and older (2). Likewise, in a population of older adults (60–69 years old) in Norway, having complex multimorbidity was found to be strongly associated with the need for IADL assistance (RR = 1.80; 95% CI, 1.58–2.04) after a follow-up of 11 years (54). On the other hand, in older adults (≥60 years old) in India, all combinations of multimorbidity were associated with more significant functional limitations (46). However, people with a combination of high blood pressure, arthritis, and depressive symptoms had a more considerable disability in ADLs-IADLs than with any other disease combination (55).

It has been found that sex is a determinant of multimorbidity because women live longer than men, but they live in poorer health conditions (19, 56). These differences have also been attributed to gender norms that limit health behavior, the search for healthcare services, and biological aspects that influence the clinical expression of certain diseases (19, 56). Globally, multimorbidity (in all age groups and by all types of disease) has been estimated to be 39.4% for women (95% CI, 36.4–42.2) and 32.8% for men (95% CI, 30.0–35.6) (4). Nevertheless, the prevalence of multimorbidity among adults aged ≥20 years is about the same for both sexes in some countries, such as Japan (57). In the present study, the prevalence of cardiometabolic multimorbidity among women was found to be 7.2 percentage points higher than among men. This gap was almost negligible for younger adults (0.5 percentage points) but was more expansive for the age group of 40–59 years (10.5 percentage points) and the age group of ≥60 years (17.3 percentage points). These results suggest that public policies need to consider the sex gaps to reduce the burden of health inequities (58).

In low- and middle-income countries, the most common multimorbidity patterns are those consisting of cardiometabolic causes such as high blood pressure, acute myocardial infarction, angina pectoris, heart failure, stroke, T2D, hyperlipidemia, and obesity (23). In this study, obesity was the common denominator in all cardiometabolic multimorbidity clusters, regardless of age group. Mexico has one of the highest rates of obesity in the world (body mass index [BMI] ≥30 kg/m^2^), with a prevalence of 36.1% among adults aged ≥20 years (59). In addition, Class 3 obesity had the most significant increase in the country in recent years, even more than the increase observed for overall obesity and for Class 1 and Class 2 obesity (59). Therefore, programs and strategies intended to address cardiometabolic multimorbidity should focus on the reduction of obesity and establish it as a cross-sectional goal.

Mexico has a national program for cardiometabolic diseases (obesity, T2D, hypertension, and dyslipidemias) with 5 priority objectives, 8 strategies, and 26 specific actions. The objectives focus on improving care processes, unifying information systems, increasing professional competencies, ensuring governance in the national health system, and incorporating mechanisms for the timely identification of complications arising from cardiometabolic diseases (30). However, the proposal has a medium- and long-term scope to be fulfilled, so it is still necessary to continue promoting and improving this initiative.

## 5 Conclusion

Understanding the behavior of cardiometabolic multimorbidity at different stages of adulthood is a resource that could be used to design and implement intervention strategies. Such strategies should correspond to the population’s sociodemographic, health, and lifestyle characteristics and the specific disease patterns of each age group. Prevention of cardiometabolic diseases is an urgent goal in Mexico. Nevertheless, for people who already have cardiometabolic multimorbidity, it is necessary to delay progression and avoid complications in the early stages through comprehensive management focused on each person’s needs. Health interventions should address cardiometabolic multimorbidity and its relationship with depressive symptoms and functional limitations throughout the life course.

## 6 Limitations

The limitations of this study were as follows: (1) The data were cross-sectional, so it was impossible to determine the factors that lead to multimorbidity or the time at which adults developed the diseases. (2) All data used herein are self-reported, and no objective measurements of participants were available. However, most studies calculating multimorbidity at the population level are based on studies with similar characteristics to ENSANUT (3, 33). This is because self-reporting has been identified as a valuable proxy for estimating the prevalence of multimorbidity. (3) This analysis only considered cardiometabolic diseases from a limited list included in the survey. We suggest that other studies explore other combinations of chronic diseases, including chronic kidney disease (CKD) and cancer. (4) The higher prevalence of multimorbidity among women could be related to their greater use of health services, but we were unable to support this argument with the data source used in this manuscript. (5) Given the nature of the data, it was also not possible to establish which type of multimorbidity combination was more severe and had worse health outcomes. We are aware that not all cardiometabolic multimorbidity clusters affect individuals equally.

## Data Availability

The original contributions presented in the study are included in the article/Supplementary material, further inquiries can be directed to the corresponding author.
