# Supplementary material for: Cardiometabolic multimorbidity in Mexican adults: a cross-sectional analysis of a national survey
Source: Front Med (Lausanne). 2024 Sep 3;11:1380715. doi: 10.3389/fmed.2024.1380715 (PMC11405330; doi:10.3389/fmed.2024.1380715)
Supplement: Supplementary file 1 [file Table_1.DOCX]

Supplementary Table S1. Factor and eigenvalue for age groups

| **Age groups** | **Factor** | **Eigenvalue** |
| --- | --- | --- |
| 20-39 | Factor1 | **2.625** |
|  | Factor2 | **1.148** |
|  | Factor3 | 0.392 |
|  | Factor4 | 0.096 |
|  | Factor5 | -0.098 |
|  | Factor6 | -0.117 |
|  | Factor7 | -0.195 |
|  | Factor8 | -0.223 |
| 40-59 | Factor1 | **2.821** |
|  | Factor2 | **1.199** |
|  | Factor3 | 0.324 |
|  | Factor4 | 0.118 |
|  | Factor5 | -0.035 |
|  | Factor6 | -0.114 |
|  | Factor7 | -0.163 |
|  | Factor8 | -0.241 |
| 60+ | Factor1 | **2.456** |
|  | Factor2 | **1.297** |
|  | Factor3 | 0.275 |
|  | Factor4 | 0.024 |
|  | Factor5 | 0.001 |
|  | Factor6 | -0.112 |
|  | Factor7 | -0.193 |
|  | Factor8 | -0.220 |
